# Feasibility and acceptability of peer-delivered interventions using mHealth for PrEP services among adolescent girls and young women in DREAMS program in Botswana

**DOI:** 10.1080/16549716.2023.2231256

**Published:** 2023-07-18

**Authors:** Marie-Claude C. Lavoie, Lillian Okui, Natalia Blanco, Kirsten Stoebenau, Jessica F. Magidson, Gadzikanani Gokatweng, Kaizer Ikgopoleng, Manhattan E. Charurat, Ndwapi Ndwapi

**Affiliations:** a Division of Global Health Sciences, Department of Epidemiology and Public Health, University of Maryland School of Medicine, Baltimore, MD, USA; b Institute of Human Virology, University of Maryland School of Medicine, Baltimore, MD, USA; c Center for International Health Education and Biosecurity (Ciheb), University of Maryland School of Medicine, Baltimore, MD, USA; d Center for International Health, Education, and Biosecurity (Ciheb), Maryland Global Initiative Corporation, University of Maryland Baltimore, Gaborone, Botswana; e Department of Behavioral and Community Health, University of Maryland School of Public Health, College Park, MD, USA; f Department of Psychology, University of Maryland, College Park, MD, USA; g Botswana-University of Maryland School of Medicine Health Initiative (Bummhi), Gaborone, Botswana

**Keywords:** Pre-exposure prophylaxis, HIV prevention, Botswana, adolescent girls, peer interventions, mhealth

## Abstract

**Background:**

Adolescent girls and young women accounted for 25% of all new HIV infections despite representing only 10% of the population in Sub Saharan Africa. PEPFAR has launched the Determined, Resilient, Empowered, AIDS-free, Mentored, and Safe (DREAMS) initiative, a comprehensive HIV prevention program including PrEP services. Among adolescent girls and young women, PrEP adherence is currently sub-optimal. Tailored strategies for adolescent girls and young women to improve access and use of PrEP delivery are urgently needed to maximise its potential. Recommended interventions include peer-delivered interventions using mobile technology. However, data on the feasibility and acceptability of this approach is limited for SSA.

**Objectives:**

We assessed the feasibility and perceived acceptability of providing mHealth peer-delivered interventions to support PrEP services among adolescent girls and young women in Botswana.

**Methods:**

This cross-sectional study included HIV-negative women aged 18–24 years old seeking health services at DREAMS-supported facilities. Participants completed a survey assessing the feasibility and perceived acceptability of the mHealth peer-delivered interventions, which included the Acceptability of Intervention Measure (AIM). Descriptive analyses were performed.

**Results:**

A total of 131 participated in the study. Overall, 89% owned a mobile phone (feasibility). There was no difference in cell phone ownership between participants from rural and urban settings. Among participants, 85% reported interest in participating in a mHealth peer-delivered intervention if it was available to them. Regarding perceived acceptability for mHealthpeer support groups for PrEP, the average score on the AIM was 3.8 out of 5 (SD = 0.8).

**Conclusion:**

mHealthpeer-delivered interventions appear to be feasible and perceived acceptable among adolescent girls and young women in Botswana. This modality should be incorporated into PEPFAR’s programmatic toolkit of implementation strategies to improve PrEP services.

## Background

HIV/AIDS is the leading cause of death among adolescent girls and young women aged 15–24 years in sub-Saharan Africa (SSA) [1]. In 2020, adolescent girls and young women accounted for 25% of all new HIV infections, despite representing only 10% of the population in SSA [2]. In response to this situation, the United States President’s Emergency Plan for AIDS Relief (PEPFAR) has launched the Determined, Resilient, Empowered, AIDS-free, Mentored, and Safe (DREAMS) initiative across 15 countries, including Botswana. The DREAMS initiative includes a core package of interventions including pre-exposure prophylaxis (PrEP) services [3]. Daily oral PrEP has been shown to decrease the risk of HIV acquisition by ≥ 90% [4,5]. However, among adolescent girls and young women, PrEP adherence is currently sub-optimal [6–9]. Tailored strategies for adolescent girls and young women to improve PrEP services delivery are urgently needed to maximise the potential of PrEP.

Peer-delivered interventions have been used for various health conditions, including HIV treatment, contraceptives use and mental disorders [10–13]. Peer-delivered interventions can be defined as enlisting individuals who are similar to them in terms of demographic or social characteristics to promote and sustain health-related behaviours and can be offered through 1:1 or group sessions [14,15]. For adolescents and young adults living with HIV, peer-delivered interventions have been shown to improve health outcomes along the HIV treatment care continuum [16,17].

In recent years, especially within the context of COVID, which impacted in-person services due to movement restrictions, alternative service models for HIV prevention and treatment have been recommended including using mobile technology (mHealth) [18]. mHealth is defined as using mobile phone technology to improve the delivery of health services [19]. Despite this recommendation, the feasibility and perceived acceptability of peer-delivered interventions using mHealth (peer-delivered interventions delivered through mobile technology) remain limited, especially for PrEP among adolescent girls and young women in SSA. PEPFAR recommends that DREAMS health facilities implement technology-based peer support for PrEP (e.g. WhatsApp groups) while emphasising the critical need to evaluate these interventions, a major implementation gap [20]. A few quantitative studies have rigorously evaluated peer-delivered interventions on PrEP outcomes among adolescent girls and young women [21,22]. Peer-delivered interventions using mobile technology may experience challenges due to intermittent electricity and connectivity, data access and affordability. The aim of this study was to assess the feasibility and perceived acceptability of providing peer-delivered interventions using mHealth to support the use of PrEP from the perspectives of adolescent girls and young women to inform the development and implementation of future mHealth interventions for adolescent girls and young women in Botswana.

## Methods

### Study design and setting, and participants

We conducted a cross-sectional survey using a convenient sample of four facilities implementing the DREAMS initiative in Gaborone (*n* = 2) and Kweneng East districts (*n* = 2) from June 2022 to August 2022. In Gaborone, 12 facilities offered the DREAMS initiative while five offered DREAMS in Kweneng East districts. HIV prevalence is estimated at 11% in Gaborone (urban setting) and 18.7% in Kweneng East (rural) [23] In Botswana, the prevalence of HIV among women aged 20–24 years is 6.7% compared to 2.7% among men of the same age group [23]. Eligible participants included HIV-negative women aged 18–24 years old who sought health services at DREAMS supported facilities. At each clinic, a trained data collector approached eligible women during clinic hours during the study period, provided information about the study, and sought consent for individuals who were interested in participating in the study.

### Measures

The 15-min tablet-based survey included questions about the feasibility and perceived acceptability of using mHealth peer support groups for PrEP services. Feasibility, the extent to which an intervention, can be successfully implemented within a setting [24], was measured by assessing the proportion of adolescent girls and young women who own a cell phone, including a smartphone. For the perceived acceptability of the mHealth peer-delivered interventions, we used the validated Acceptability of Intervention Measure (AIM) developed by Weiner and colleagues [25]. AIM includes four questions answered using a scale from 1 to 5 (1= completely disagree to 5=completely agree) and has been used in the context of sub-Saharan Africa [26]. No specialised training is needed to administer or interpret the results. In addition, the survey also included questions about suggested implementation strategies to support the delivery of PrEP services. Demographic data and location of facilities (urban and rural) were collected.

### Data collection procedures and analysis

Consenting participants completed the survey in Setswana or English based on preference. The survey included closed- and open-ended questions. The research team trained the survey interviewers on data collection tool, ethics and data quality, security and confidentiality.

We completed a descriptive analysis using means and standard deviation (SD) for continuous variables. We used proportions and calculated chi-square or Fisher's exact test for categorical variables to compare by location of facilities (rural or urban). Cronbach’s Alpha statistic was estimated to determine the reliability of the AIM score [27].

## Results

### Participants demographics

Among 138 adolescent girls and young women who were asked to participate, 131 agreed and provided consent (94.9%). The mean age was 20.5 years old (SD 1.8). Overall, 54.2% were from urban areas (n = 71/131), 67.2% completed secondary school (n = 88), 35.9% attended school in the last 12 months (n = 47), and 90.8% were single (n = 119). Level of education, occupation in the last 12 months and marital status differed significantly between adolescent girls and young women living in rural areas compared to urban (*p* value < .05) (Table 1).

**Table 1.** Demographic characteristics among adolescent girls and young women participants.

Characteristics	Total n (%) N = 131	Urban n (%) n = 71	Rural n (%) n = 60	P-value
**Age (years), mean – SD**	20.5 (1.8)	20.6 (1.9)	20.3 (1.7)	.32
**Level of education**				<.01*
Primary	2 (1.5)	2 (100.0)	0 (0)	
Secondary	88 (67.2)	40 (45.5)	48 (54.6)	
University	38 (29.0)	28 (73.7)	10 (26.3)	
Declined to answer	3 (2.3)	1 (33.3)	2 (66.7)	
**Occupation for last 12 months**				<.01*
Paid work	23 (17.6)	11 (47.8)	12 (52.2)	
Going to school/study	47 (35.9)	38 (80.9)	9 (19.1)	
Looking for work	38 (29.0)	10 (26.3)	28 (73.7)	
Too ill to work	1 (0.8)	0 (0)	1 (100.0)	
Handicapped/cannot work	1 (0.8)	0 (0)	1 (100.0)	
Household work/childcare	8 (6.1)	4 (50.0)	4 (50.0)	
Other (specify)	10 (7.6)	7 (70.0)	3 (30.0)	
Declined to answer	3 (2.3)	1 (33.3)	2 (66.7)	
**Marital status**				<.02*
Single	119 (90.8)	62 (52.1)	57 (47.9)	
Married	2 (1.5)	2 (100.0)	0 (0)	
Cohabiting	6 (4.6)	6 (100.0)	0 (0)	
Declined to answer	4 (3.0)	1 (25.0)	3 (75.0)	

*Fisher's exact test.

### Feasibility, preferences and acceptability

With regard to the feasibility of peer-delivered interventions through mHealth, 88.6% owned a mobile phone and among adolescent girls and young women who owned a cell phone, 93.1% had a smartphone. Ninety-seven percent communicated using WhatsApp in the last 12 months. No difference was observed between rural and urban groups (Table 2).

**Table 2.** Feasibility and perceived acceptability of peer-delivered interventions using mHealth.

Feasibility	Overall *N* = 131 n %	Urban (*n* = 71)	Rural (*n* = 60)	*P* value*
Own a mobile phone	116 (88.6)	62 (87.3)	54 (90.0)	.23
Own a smartphone^€^	108 (93.1)	58 (93.5)	50 (92.6)	.55
Communicate using an application such as WhatsApp in the last 12 months^€^	112 (96.6)	58 (93.6)	54 (100)	.31
Acceptability	Overall *N* = 129^&^ Mean (SD)	Urban n = 70 Mean (SD)	Rural n = 59 Mean (SD)	*P* value**
Acceptability Intervention Measure (AIM)	3.84 (0.84)	3.88 (0.85)	3.81 (0.84)	.66

^€^Among those who reported owning a cellphone.

^&^Excluded two participants who did not answer this section of the survey.

*p value estimated using chi-square.

**p values estimated using t-test.

Overall, 26.7% (*n* = 35/131) had previously participated in peer-delivered interventions using mobile technology offered by the clinic. In terms of interest in peer-delivered interventions through mHealth, 84.7% (*n* = 111) reported being interested if it was available to them by the facility. There was no difference between participants seeking HIV services in urban and rural locations. Among individuals who indicated their preferences for the type of peer support group (*n* = 85), 44 (51.8%) preferred a hybrid model (combination of in-person and mHealth), 26 (30.6%) through mobile technology, 13 (15.3%) in-person at the facility and 2(2.4%) in-person in the community. There was no significant difference between adolescent girls and young women living in rural compared to urban settings (Table 3).

**Table 3.** Experience and interest towards peer-delivered interventions using mHealth.

Variable	Overall N = 131 n (%)	Urban N = 71 n (%)	Rural N = 60 n (%)	P value
**Previous experience with peer-delivered interventions using mHealth**				
Yes	35 (26.7)	17 (23.9)	18 (30.0)	.45*
No	89 (67.9)	49 (69.0)	40 (66.7)	
Don’t know	3 (2.3)	3 (4.2)	0 (0)	
Declined	4 (3.1)	2 (2.8)	2 (3.3)	
**Interest in participating in peer-delivered interventions using mHealth**				
Yes	111 (84.7)	59 (83.1)	52 (86.7)	.92*
No	10 (7.6)	6 (8.4)	4 (6.7)	
Don’t know	6 (4.6)	4 (5.6)	2 (3.3)	
Declined	4 (3.1)	2 (2.8)	2 (3.3)	
**Preferences for the type of peer-delivered intervention**^€^				
Using mobile technology	26 (30.6)	16 (61.5)	10 (38.5)	.78*
In-person at facility	13 (15.3)	9 (69.2)	4 (30.8))	
In-person at community	2 (2.4)	1 (50.0)	1 (50.0)	
Hybrid (in-person and mobile)	44 (51.8)	24 (54.5)	20 (45.5)	
**Implementation strategies suggested by adolescent girls and young women to optimise use of PrEP^&^**				
Peer-delivered interventions (in-person, mobile or hybrid)	89 (67.9)	54 (76.1)	35 (58.3)	.03
Short waiting time for PrEP refill pick up	81 (61.8)	47 (66.2)	34 (56.7)	.26
PrEP refill at the community	50 (38.2)	30 (42.3)	20 (33.3)	.30
SMS or phone call reminders for PrEP refill	103 (78.6)	55 (77.5)	48 (80.0)	.72
Dedicated safe place at the clinic for PrEP services	88 (67.2)	38 (53.5)	50 (83.3)	<.01

*Fisher’s exact test ^€^Only among who indicated their preferences for the type of peer support group ^&^Participants could select more than one option.

Regarding perceived acceptability for peer support groups through mHealth for PrEP, the average score on the AIM was 3.8 (SD = 0.8) out of 5. No significant difference is observed in the average score between urban and rural residents. The AIM Cronbach’s alpha was 0.95, suggesting high internal consistency between the AIM’s questions. Across AIM questions, 80% or more of participants either agree or completely agree with the AIM statement. When asked about preferred strategies to support PrEP services, 78.6% (*n* = 103/131) of participants selected SMS or phone call reminders for PrEP refills, 67.9% (*n* = 89) peer-delivered interventions (in-person, mobile or hybrid), and 67.2% (*n* = 88) dedicated safe place at the clinic for PrEP services were among the top preferred. A higher proportion of participants in urban facilities selected peer-delivered interventions than those from rural facilities (76.1% vs. 58.3%, *p* = 0.03). SMS or phone call reminders for PrEP refills and a dedicated safe place in the clinic for PrEP services were also highly selected within the participant’s top three interventions (Table 3). Rural participants selected the need for a dedicated safe place significantly more than urban facilities (83.3% vs. 53.5%).

## Discussion

In this cross-sectional study, we found a high proportion of adolescent girls and young women who had access to mobile technology irrespective of the setting, which is consistent with a study from Kenya including adolescent girls and young women in DREAMS program [28] and aligned with a global trend [29]. The increasing accessibility of mobile technology and the internet can be leveraged to increase access to health services to improve health outcomes. A high proportion of participants expressed an interest in receiving peer-delivered interventions using mHealth. Our results are similar to other studies that found peer-delivered intervention groups delivered through mobile phone are acceptable to young individuals [30,31].

Most research on peer-delivered interventions using mHealth includes people living with HIV with less emphasis on HIV prevention [32,33]. For PrEP, mobile technology has been mostly limited to training staff remotely, PrEP demand creation and SMS reminders [34]. Touger et al., (2019) conducted a review on technology-based intervention for PrEP [35]. However, this review focuses primarily on telehealth programmes without information on peer-delivered intervention for PrEP [35] leaving gaps in its implementation and effectiveness to support the effective use of PrEP, such as the incorporation of peer-delivered interventions. As most adolescent girls and young women discontinue PrEP within the first month across geographical settings [9,36,37], it is critical to identify and evaluate new implementation strategies intervening during this critical window to optimise the full potential of PrEP, one of the most effective strategies to prevent HIV acquisition. Although peer support for PrEP services was ranked high across groups, priority preferences for implementation strategies differed between rural and urban groups, highlighting the importance of context and engaging users when developing interventions.

The successful integration of mobile technology for health services, including peer-delivered interventions, will hinge on several determinants. Implementation considerations are needed to ensure privacy, confidentiality and data security [38]. This is especially relevant for PrEP services as physical and virtual environments lacking privacy and confidentiality may discourage adolescent girls and young women from seeking PrEP services due to HIV and sexual stigma associated with PrEP [39,40]. The integration of peers into the health system for HIV prevention is also another important consideration for its effectiveness and sustainability. For HIV treatment, evidence from Botswana suggest that peers can be successfully embedded within the health system with appropriate planning, management, coordination and resources [16].

The study is subject to limitations. The survey was limited to four clinics selected using convenience sampling, which may not be generalisable nationally. However, we included adolescent girls and young women at risk for HIV acquisition (eligibility criteria to be enrolled into DREAMS), a group relevant for peer-delivered intervention using mHealth. The study sample included a similar proportion of adolescent girls and young women who were single (90.8% vs 85.5%) and had a slightly higher proportion of individuals who had completed high school (67.2% vs 59.5%) compared to the overall population of adolescent girls and young women seeking services at DREAMS supported facilities during the study period. The survey consisted of mostly closed-ended quantitative questions; qualitative data would have provided more in-depth data from adolescent girls and young women. Despite AIM used in different settings including sub-Saharan Africa [26], this tool has been validated in high-income countries. The study strengths included the inclusion of adolescent girls and young women from rural and urban settings to include diverse perspectives with a response rate of 95%.

In conclusion, according to adolescent girls and young women, peer-delivered interventions using mobile technology appear to be feasible and perceived as acceptable across adolescent girls and young women in Botswana. This modality should be considered in the PEPFAR’s programmatic toolkit of implementation strategies to improve PrEP services. Future studies are warranted to assess the effect of peer-delivered interventions on PrEP continuation.

## Paper context

PrEP is highly effective against HIV acquisition. Despite the availability of PrEP, most adolescent girls and young women) discontinued PrEP within the first month. Tailored interventions are urgently needed. Peer-delivered through mobile technology could enhance PrEP adherence. However, little is known about the acceptability and feasibility of this intervention in Botswana. Our findings suggest that peer-delivered intervention through mHealth is feasible and acceptable to adolescent girls and young women and should be evaluated to assess its impact.
